# Phytonutrients and their neuroprotective role in brain disorders

**DOI:** 10.3389/fmolb.2025.1607330

**Published:** 2025-09-02

**Authors:** Krishnendu Adhikary, Krishnendu Ganguly, Riya Sarkar, Md. Abubakar, Pradipta Banerjee, Prithviraj Karak

**Affiliations:** ^1^ Department of Medical Laboratory Technology, Paramedical College Durgapur, Durgapur, West Bengal, India; ^2^ Department of Medical Laboratory Technology, Dr. B. C. Roy Academy of Professional Courses, Durgapur, West Bengal, India; ^3^ Department of Pharmacology and Toxicology, National Institute of Pharmaceutical Education and Research, Guwahati, Assam, India; ^4^ Deccan School of Pharmacy Darussalam, Hyderabad, Telangana, India; ^5^ Department of Surgery, University of Pittsburgh, Pittsburgh, PA, United States; ^6^ Department of Physiology, Bankura Christian College, Bankura, West Bengal, India

**Keywords:** cognitive impairment, schizophrenia, neuroinflammation, phytochemicals, medicinal plants

## Abstract

In the twenty-first century, cognitive impairment is a significant health problem. Function is substantially impaired by a number of neuropsychiatric and neurodegenerative diseases, such as Parkinsonism, schizophrenia, major depressive disorder, Alzheimer’s disease and other types of cognitive impairment, cerebrovascular disabilities, seizure-related disorders, and brain traumas. Over time, a number of chemical messengers and signaling molecules have been identified as potential targets for treatment, and tests have been performed against these targets using both conventional and novel chemicals. Phytochemicals derived from medicinal plants are essential for preserving the chemical balance of the central nervous system because they change the activity of major inhibitory receptors that receive neurotransmitters. Many herbs have been used in conventional medicine to treat cognitive problems. Although the presence of receptors that are responsible or transporters for compounds called polyphenols and other phytochemicals in brain regions remains to be determined, multiple target substances seem to be a promising class of treatment options for treating disorders with multifactorial origins. Additional studies suggest that flavonoids possess significant anti-inflammatory properties in the brain, making them a promising therapeutic option for conditions such as ischemic or hemorrhagic stroke, as well as chronic neuroinflammatory disorders like Parkinson’s and Alzheimer’s disease. This review highlights how phytochemicals contribute to the protection against brain disorders and explores the underlying mechanisms involved in their action. It also emphasizes the core biological processes, providing deeper insight into the therapeutic potential of phytochemicals in the treatment of neurological conditions.

## 1 Introduction

Plants provide essential elements, including vitamins, minerals, and bioactive compounds that are necessary for maintaining human health (Zhao et al., 2021). Phytonutrients are naturally occurring plant components that have garnered a lot of interest due to their numerous health benefits (Wang et al., 2022a). When included in the human diet, these secondary metabolites—originally aiding plants by providing flavor, color, and defense against infections—also offer remarkable medicinal benefits (Smith and Lee, 2023). With the increasing incidence of chronic conditions including malignancies, heart disease, and disorders of the brain, scientists are focusing increasingly on micronutrients’ potential as a natural and sustainable means of disease prevention and health promotion (Patel P. et al., 2023). The human nervous system is an extremely intricate organ that controls emotions, muscular control, and cognitive processes (Kumar et al., 2021a). However, age, factors related to the environment, and lifestyle choices are the main causes of the growing prevalence of neurological illnesses such as depression, multiple sclerosis (MS), Parkinson’s disease (PD), and Alzheimer’s disease (AD) among the world’s population (Nguyen et al., 2022). Although the goal of traditional pharmaceutical therapies is to control symptoms, they frequently fall short of stopping the course of the disease and can have adverse consequences (Smith and Lee, 2023). Because of their anti-inflammatory, neurogenic, and antioxidant qualities, scientists have thus looked to phytonutrients—naturally occurring vegetable compounds—as possible neuroprotective medicines (Patel P. et al., 2023). The function of phytonutrients in shielding neurons from free radical damage and neurodegeneration has been brought to light by recent advances in neurobiological and nutritional neuroscience (Wang and Zhao, 2021). Numerous studies have looked at the possible ability of flavonoids that are polyphenols, and carotenoids to enhance cognitive function, reduce neuroinflammation, and halt the formation of amyloid plaque, a hallmark of Alzheimer’s disease (Johnson et al., 2008). One of the most promising phytonutrients is curcumin, which is isolated from turmeric (*Curcuma longa*). Curcumin has been shown to change signaling pathways that control synaptic plasticity, raise the amount of neurotrophic factor from the brain (BDNF), a protein essential for neuron survival and mental performance, and reduce β-amyloid accumulation. Similarly, resveratrol, a polyphenol found in grapes and red wine, has demonstrated neuroprotective qualities by activating sirtuin receptors (SIRT1), which are in charge of mitochondrial functioning and cellular aging (Lee and Kim, 2024). Flavonoids found in green tea—particularly quercetin and epigallocatechin gallate (EGCG)—have been found to support the growth of new neurons in the hippocampus, protect dopaminergic neurons from damage linked to Parkinson’s disease, and influence gut-brain interactions that impact mental health.

Additionally, two carotenoids found in leafy greens, lutein and zeaxanthin, have been linked to decreased age-related cognitive decline and enhanced cognitive resilience (Zhao and Chen, 2024). Another emerging area of research in brain health is the role of the gut bacteria in protecting neurons. Recent studies have shown that gut bacteria and phytonutrients combine to produce neuroactive metabolites that can improve mental health and lessen neuroinflammation (Zhang and Li, 2023). This link, known as the “gut-brain connection,” highlights the importance of dietary phytonutrients in maintaining neuronal homeostasis and preventing cognitive aging. Although phytonutrients show promising potential for supporting brain health, several research challenges remain to be addressed. Many phytonutrients, such as resveratrol and curcumin, have limited absorption, high metabolism, and low solubility, which limits their ability to target the brain and spinal cord. To improve BBB permeability and bioactivity, cutting-edge nanotechnology-based delivery methods such as nanoparticles of lipids, liposomes and phytosomal formulation are now being investigated. Long-term human studies are required to demonstrate the neuroprotective benefits of phytonutrients in a variety of groups, even if preclinical research and small-scale experimental studies yield encouraging results (Patel P. et al., 2023). To confirm their safety and effectiveness, large randomized controlled experiments (RCTs) using biomarkers and uniform doses are necessary. Rarely do phytonutrients function alone. It is yet unclear how they work in concert with mineral content, vitamins, and other dietary elements. Future studies should examine how phytonutrient combinations, such flavonoids and omega-3 fatty acids, might improve neuroprotection and cognitive advantages (Johnson et al., 2008). Although the gut-brain axis has drawn more attention, little is known about how phytonutrients are converted into bioactive neuroprotective chemicals by microbial metabolism. Examining individualized diet regimens based on the composition of the gut microbiota may be necessary to maximize the benefits of phytonutrients for psychological health (Zhang and Li, 2023). The neuroprotective potential of many foods high in phytonutrients may be diminished by oxidation, enzymatic breakdown, and heat preparation. These bioactives may be preserved for use in functional foods and nutritional supplements by creating innovative food processing methods such as extraction by cold and fermentation.

Novel, natural treatment approaches are required due to the increasing incidence of mental health and neurodegenerative diseases (Patel P. et al., 2023). With their neurogenic, antioxidant, and anti-inflammatory qualities, phytonutrients provide exciting opportunities for improving cognitive function and brain health (Wang et al., 2022b). Our knowledge of their processes has grown as a result of recent developments in nutritional neurological science, nanotechnologies and microbiome research (Zhang et al., 2021). However, converting phytonutrient research into useful therapeutic applications for neurological diseases requires tackling important issues including bioavailability, extensive clinical validation, and combinatorial dietary interactions (Lee and Kim, 2024). Unlocking the full promise of phytochemicals in brain health will need future research centered on customized diet and innovative delivery systems (Kumar et al., 2025a; Kamli et al., 2022).

## 2 Phytonutrients impact on brain health

Phytonutrients, also known as phytochemicals, are naturally occurring compounds found in a wide variety of plant-based foods, including vegetables, fruits, whole grains, legumes, nuts, seeds, and cereals (Singh et al., 2023a). While they are not classified as essential nutrients like vitamins or minerals, phytonutrients play a significant role in promoting health and preventing disease (Poles et al., 2021a; Kamli et al., 2022). These bioactive compounds, such as flavonoids, carotenoids, and polyphenols, possess antioxidant, anti-inflammatory, and neuroprotective properties that are particularly beneficial for brain health (Wang et al., 2022c; Hussain et al., 2013). By reducing inflammation and oxidative stress in the brain, phytonutrients help protect neurons from damage, support the formation of new neural connections, and improve communication between brain cells (Zhao and Chen, 2024). As a result, they have been linked to enhancements in cognitive functions such as memory, learning, attention, and decision-making (Lee et al., 2023). Additionally, certain phytonutrients influence the production and regulation of neurotransmitters like serotonin and dopamine, which are key chemicals involved in mood regulation and emotional wellbeing (Kumar et al., 2025b). Regular consumption of a diet rich in diverse plant-based foods ensures a steady intake of these beneficial compounds, thereby supporting not only long-term brain health but also contributing to better mental clarity, mood stability, and overall psychological resilience (Poles et al., 2021a; Hussain et al., 2013).

### 2.1 Anti-inflammatory effects of phytonutrients

Numerous naturally occurring compounds found in plants, known as plant-based nutrients, have the potential to lower inflammation in the brain and shield brain cells from harm. This is significant since a variety of neurological disorders, such as depressive disorders, Alzheimer’s disease, and Parkinson’s disease, and cognitive decline, is associated with chronic inflammation in the brain (Davinelli et al., 2016; Puri et al., 2022). Additionally, they contain potent anti-inflammatory properties that are critical for preserving and safeguarding brain health.

### 2.2 Antioxidant properties and reduction of oxidative stress

Flavonoids, carotenoids and phytochemicals are examples of phytonutrients that include antioxidants that help the body fight off harmful free radicals. The free radicals, those are very damaging molecules that may damage cells and tissue, including those lining the brain, are the source of oxidative stress. Oxidative stress is linked to a number of neurodegenerative illnesses, such as Parkinson’s, Alzheimer’s, and general cognitive decline (Gupta and Prakash, 2014). Oxidative stress in the brain can damage neurons and the blood-brain barrier, leading to neurodegenerative diseases and reduced cognitive function. The neuroprotective effects of phytonutrients help reduce this damage by various signalling process (Singh, 2019).

#### 2.2.1 Enhancing mitochondrial function

The cell’s energy-producing organelles, the mitochondria, can be hampered by oxidative stress. By protecting mitochondria, phytonutrients can help brain cells produce energy at their best, maintaining proper neuronal function, enhancing synaptic activity, and supporting cognitive processes such as memory, learning, and mood regulation. (Zhang et al., 2019).

#### 2.2.2 Reducing inflammation

One feature of neurodegenerative diseases is persistent inflammation. Rahman et al. (2020) claim that phytonutrients like curcumin from turmeric and resveratrol from grapes help reduce inflammation in the nervous system while also maintaining cognitive function.

#### 2.2.3 Promoting neuronal repair and regeneration

According to Poles et al. (2021b), certain micronutrients have been demonstrated to promote the synthesis of brain-derived neurotrophic factor (BDNF), a protein that aids in the development and survival of neurons and promotes brain regeneration and repair. By enhancing BDNF levels, these micronutrients may improve synaptic plasticity, support cognitive performance, and potentially slow the progression of neurodegenerative conditions such as Alzheimer’s and Parkinson’s disease (Puri et al., 2022).

### 2.3 Role in enhancing brain plasticity and neurogenesis

Enhancing brain flexibility and neurogenesis—two processes that are critical for memory, learning, cognitive function, and general brain health—is made possible by phytonutrients. The ways in which phytonutrients support these processes are broken down as follows:

#### 2.3.1 Brain plasticity

Brain plasticity, sometimes referred to as neuroplasticity, is the brain’s ability to reorganize and form new neural connections in response to circumstances, education, and environmental changes. Maintaining cognitive ability over time, healing from brain damage, and adjusting to new circumstances all depend on neuroplasticity (Fujimura et al., 2002; Kolb and Whishaw, 1998). Brain plasticity is improved by phytonutrients through:

#### 2.3.2 Stimulating brain-derived neurotrophic factor (BDNF)

Some refer to BDNF, a protein that supports neuronal development, survival, and differentiation, as “fertilizer for the brain.” It has been demonstrated that certain phytonutrients, such as curcumin (found in turmeric), resveratrol (found in grapes and berries), and flavonoids (found in berries and gloomy chocolate), raise BDNF levels, which in turn promote neuroplasticity by promoting the development of new neural pathways and the fortification of preexisting ones (Hannan et al., 2020; Kolb and Whishaw, 1998).

#### 2.3.3 Enhancing synaptic plasticity

For example, flavonoids from food items like blueberries have been associated to enhanced plasticity of synaptic connections, which helps the nervous system adapt and learn novel information more efficiently (Fujimura et al., 2002). Additionally, plants can enhance plasticity of synaptic cells, which is the strengthening and shrinking of synapses and is essential for memory and learning.

#### 2.3.4 Protecting neurons from stress and damage

Neuroplasticity may be impeded by inflammation and oxidative stress. By lowering oxidative stress, phytonutrients shield neurons from harm and help the brain adjust and reorganize. It has been demonstrated that polyphenols, such as those in green tea, lessen oxidative neuronal damage, promoting long-term neuroplasticity (Nath et al., 2023).

### 2.4 Neurogenesis

Neurogenesis is the process by which neural stem cells are used to produce new neurons. The hippocampus, a part of the brain involved in learning and memory, is where this process mostly takes place. Diet and lifestyle can have an impact on neurogenesis, which is essential for mental agility and emotional control (Nath et al., 2023; Sharma et al., 2024). Phytonutrients promote neurogenesis through:

#### 2.4.1 Increasing neural stem cell proliferation

Numerous studies have shown that phytonutrients can encourage neural stem cells—the cells that develop into new neurons—to proliferate. In animal models, flavonoids such as curcumin and those in apples and blueberries have been demonstrated to promote the growth of brain stem cells. According to Hussain et al. (2023), these substances stimulate signaling pathways that encourage the development of new brain cells (Sharma et al., 2024).

#### 2.4.2 Promoting hippocampal neurogenesis

The hippocampus is one part of the brain whose function is crucial for mood control, memory, and learning. Hippocampal neurogenesis is essential for cognitive function. According to research, phytonutrients like epicatechins, which are present in dark-colored chocolate and tea, and resveratrol, which is found in grapes and red wine, can increase neurogenesis in this area, improving memory as well as learning (Hussain, 2023).

#### 2.4.3 Improving neuroplasticity through neurogenesis

In order to promote flexibility and adaptive brain function, neurogenesis facilitates the synthesis of new neurons, which then integrate into the brain’s networks. Flavonoids and anthocyanins, which are present in berries like cherries and blueberries, promote neuroplasticity and neurogenesis, which improves the brain’s capacity for memory formation and learning (Basu et al., 2021; Koponen et al., 2007).

## 3 Sources of key phytonutrients and their neuroprotective mechanisms

### 3.1 Polyphenols (e.g., flavonoids, resveratrol)

A class of naturally occurring substances called polyphenols, which are widely distributed in plants, are well-known for their neuroprotective, anti-inflammatory, and antioxidant qualities. Flavonoids and resveratrol are two of the most popular and extensively researched polyphenols; they both have a major impact on cognitive performance and brain health (Khan et al., 2023a). Effects and mechanisms on brain function:

#### 3.1.1 Antioxidant activity

Polyphenols, a diverse group of naturally occurring compounds found abundantly in fruits, vegetables, tea, wine, and other plant-based foods, are renowned for their potent antioxidant properties (Wang et al., 2021a). As antioxidants, they play a critical role in neutralizing free radicals—unstable molecules that can cause cellular damage when their levels become excessive (Chen et al., 2022). In the human brain, where high oxygen consumption and lipid content make neurons particularly vulnerable, this antioxidant action is especially important (Khan M. S. et al., 2023). Free radicals can trigger a cascade of oxidative stress, a harmful process that damages cellular structures such as proteins, lipids, and DNA (Patel et al., 2024a; Pande and Akoh, 2010). Over time, such oxidative damage contributes to the onset and progression of various neurodegenerative disorders, including Alzheimer’s disease, Parkinson’s disease, and other forms of cognitive decline (Lee and Park, 2022). By scavenging these reactive oxygen species and reducing oxidative stress, polyphenols help preserve the integrity of neuronal cells and promote healthy brain aging (Zhao et al., 2025). Consequently, regular consumption of polyphenol-rich foods is believed to support cognitive function, protect against memory loss, and maintain overall neurologicalhealth (Khan M. S. et al., 2023; Pande and Akoh, 2010).

#### 3.1.2 Anti-inflammatory effects

Neurodegenerative disorders and cognitive impairment are associated with chronic inflammation. By regulating the expression of inflammatory substances including cytokines and processors (like COX-2), polyphenols lessen inflammation. This lessens the risk of inflammation-induced damage to brain tissue (Hasan et al., 2024).

#### 3.1.3 Neurogenesis stimulation

Certain polyphenols, particularly resveratrol (found in the wines and grapes), have been shown to promote neurogenesis, the process by which new neurons are formed in the brain. This is essential for memory, learning, and the recovery from brain trauma. Furthermore, neurogenesis plays a critical role in halting age-related cognitive decline.

#### 3.1.4 Modulation of brain signaling pathways

Polyphenols can alter different brain pathways of signaling, including those linked to transmitters such as dopamine, serotonin, and acetylcholine. These neurotransmitters are important in mood regulation, memory, and cognitive process (Wang et al., 2021b; Kim and Lee, 2023). According to Islam et al. (2022), polyphenols have the potential to improve dopamine signaling, which is crucial for motivation, focus, and learning, by modulating oxidative stress, enhancing synaptic plasticity, and supporting the survival of dopaminergic neurons—factors that collectively contribute to better cognitive and emotional functioning.

#### 3.1.5 Blood-brain barrier penetration

Polyphenols have the ability to cross the blood-brain barrier, a selective permeability barrier that protects the brain from harmful substances. Because of this, polyphenols can directly interact with the brain to produce their beneficial effects, including enhancing cognitive function, reducing neuroinflammation, and protecting against neurodegenerative diseases (Islam et al., 2022).

#### 3.1.6 Potential benefits in neurodegenerative diseases

I. Research indicates that polyphenols can enhance cognitive processes including memory retention, learning, and attention. For example, polyphenols found in berries, such as blueberries, have been demonstrated to enhance memory function and postpone age-related cognitive decline (Travica et al., 2020).
II. Polyphenols possess antioxidant and anti-inflammatory properties that may aid in the treatment of mood disorders. Some studies suggest that polyphenols can alleviate symptoms of depression and anxiety by stimulating the production of brain-derived neurotrophic factor (BDNF), a protein crucial for emotional regulation and neurological health.
III. Several studies suggest that polyphenols, including flavonoids, curcumin, and a substance called resveratrol could offer defense against neurodegenerative diseases such as Alzheimer’s and Parkinson’s. These compounds improve brain mitochondrial function, reduce inflammation, and decrease the accumulation of toxic proteins (such amyloid-beta in the development of Alzheimer’s disease) (Gandla et al., 2023).
IV. It has been demonstrated that some polyphenols, especially those present in cocoa and dark chocolate, enhance cerebral blood flow (Martinez et al., 2021; Smith and Lee, 2023). Better oxygen and nutrient delivery to the brain as a result of this increased blood flow may promote cognitive performance and general brain health (Hernandez et al., 2024).

### 3.2 Carotenoids (e.g., lutein, zeaxanthin)

Carotenoids are naturally occurring pigments that may be found in a variety of fruits and vegetables, including lutein and zeaxanthin. These compounds, which belong to the class of antioxidants, have been shown to have significant benefits for both cognitive function and visual health (Batool et al., 2022).

#### 3.2.1 Impact on cognition

##### 3.2.1.1 Cognitive function and memory

Recent studies have shown that higher levels of both zeaxanthin and lutein in the brain, especially in regions linked to memory and learning, are linked to improved cognitive performance, particularly in older adults. This suggests that those carotenoids may also play a role in memory and thinking ability (Batool et al., 2022).

##### 3.2.1.2 Neuroprotection and aging

As antioxidants, lutein and zeaxanthin help neutralize free radicals in the brain, which are known to contribute to neurodegeneration and age-related cognitive decline. By reducing oxidative stress, carotenoids may help protect neurons from damage and support healthy brain aging (Pawlowska et al., 2019).

##### 3.2.1.3 Improved neural communication

Lutein and zeaxanthin may also affect neuroplasticity, which is the brain’s ability to reorganize itself by forming new neural connections. This process is crucial for learning and memory.

##### 3.2.1.4 Potential benefits in neurodegenerative diseases

Carotenoids like lutein and zeaxanthin may have psychologically beneficial impacts against age-related neurodegenerative conditions like Parkinson’s disease and Alzheimer’s disease, and these are often linked to higher levels of inflammation and oxidative stress, according to Pawlowska et al. (2019).

#### 3.2.2 Impact on visual health

##### 3.2.2.1 Protection against age-related macular degeneration (AMD)

The ability of lutein and zeaxanthin to shield the eyes against macular degeneration caused by age (AMD), a major contributor to vision loss in older persons, is among its best-known advantages. According to Veselinović et al. (2021), lutein and zeaxanthin both build up in the macula located inside the eye, where they function as filters, absorbing damaging blue light and lowering oxidative stress that can destroy retinal cells.

##### 3.2.2.2 Improved contrast sensitivity and visual acuity

In low light, contrast sensitivity—the capacity to discern objects from backgrounds—is enhanced by lutein and zeaxanthin. These carotenoids improve optical acuity, particularly in low light, by filtering damaging blue light.

##### 3.2.2.3 Reduction in cataract risk

Carotenoids, namely, lutein and zeaxanthin, have been shown to protect against cataract development, which causes the eye’s lens to become hazy and impair vision (Veselinović et al., 2021).

##### 3.2.2.4 Protection against oxidative stress and inflammation

Because of its high rate of metabolism and exposure to light, the retina is especially susceptible to oxidative damage. As potent antioxidants, lutein and zeaxanthin shield the eye from damage caused by free radicals and lower inflammation. This defense aids in preserving the general health and functionality of the eyes.

### 3.3 Omega-3 fatty acids (e.g., DHA, EPA)

According to Kidd (2007), omega-3 fatty acids, particularly DHA (docosahexaenoic acid) and EPA (eicosapentaenoic acid), are vital lipids that support everything from brain structure and function to mood management and cognitive development.

#### 3.3.1 Role in supporting brain structure and function

##### 3.3.1.1 Brain development and maintenance

Particularly in the retina and the gray matter of the brain, which processes information, DHA is an essential structural element of neuronal membranes. DHA is one of the greatest prevalent fatty acids in the brain, which is really composed of around 60% fat. For appropriate cell signaling and neuronal communication, DHA aids in preserving the fluidity and flexibility of brain cell membranes (Veselinović et al., 2021).

##### 3.3.1.2 Cognitive function and memory

Omega-3 fatty acids, particularly DHA, have been shown to improve cognitive functions including retention of information, recall, and processing speed (Chen et al., 2022; Park and Lee, 2024). Research has shown that higher levels of DHA in the brain are associated with better verbal fluency, speed of processing, and memory recall skills, especially in older adults (Singh et al., 2023b).

##### 3.3.1.3 Mood regulation and mental health

Strong anti-inflammatory qualities of EPA in particular aid in lowering brain inflammation, which is connected to a number of mental health issues. Inflammation that continues in the central nervous system can cause disorders including schizophrenia, anxiety, and depression by affecting neurotransmitter activity. Mood disorders, particularly depression, are frequently treated with omega-3 fatty acids. Research has demonstrated that taking supplements of EPA and DHA helps lessen depressive symptoms; in fact, there is some indication that EPA could represent a better option for treating depression than DHA. Omega-3 fatty acids help regulate the production and functioning of neurotransmitters including serotonin and dopamine, which are crucial for mood modulation (Valenzuela et al., 2012).

##### 3.3.1.4 Brain aging and neurodegenerative diseases

Omega-3 fatty acids have been shown to slow down the rate in which cognitive decline happens in older adults. Higher DHA levels have been linked to improved cognitive performance in older adults, according to studies. According to Valenzuela et al. (2012), omega-3 fatty acids may assist maintains the integrity of the gray matter in the brain and guard against the atrophy of areas crucial for memory and cognition.

##### 3.3.1.5 Potential neuroprotective effects in aging

One of the primary roles of DHA in the brain is to maintain the structural integrityof neuronal membranes. As the brain ages, the membranes of brain cells can become more rigid, and synaptic communication can deteriorate, impairing cognitive functions. DHA helps maintain membrane fluidity, which is essential for efficient signaling and communication between neurons (Kulkarni and Dhir, 2010).

Studies show that older adults with higher levels of DHA in their blood tend to have better gray matter volume (the area involved in processing information) and white matter integrity (which connects different regions of the brain). DHA helps protect against the shrinkage of key brain regions, particularly the hippocampus, which is essential for memory and learning.

According to research, DHA and EPA may help slow down the pace of brain shrinkage, which is associated with dementia and cognitive loss and usually increases with age. Higher omega-3 fatty acid levels have been linked to slower rates of hippocampus shrinkage, the area most affected by Alzheimer’s disease, in those with moderate cognitive impairment (MCI) (Kulkarni and Dhir, 2010).

### 3.4 Curcumin

Curcumin, the main component of turmeric (Curcuma longa), has attracted a lot of attention because of its neurogenic and anti-inflammatory properties, particularly in relation to brain health and neuroprotection (Kumar et al., 2021b; Zhang and Li, 2023). This bioactive compound is widely recognized for its potent antioxidant and anti-inflammatory qualities, which are crucial for promoting neurogenesis, or the development of new neurons, and avoiding age-related brain problems (Patel et al., 2024b).

#### 3.4.1 Anti-inflammatory and neurogenic properties

##### 3.4.1.1 Increase in brain-derived neurotrophic factor (BDNF)

The protein known as brain-derived neurotrophic factor, or BDNF, promotes the development, survival, and differentiating of new neurons and is vital for synaptic plasticity, which is necessary for memory and learning. Curcumin promotes neurogenesis and improves brain function by increasing BDNF expression (Limcharoen et al., 2021). One of the primary ways curcumin stimulates neurogenesis is in this way.

##### 3.4.1.2 Activation of key signaling pathways

Numerous signaling pathways connected with the control of neurogenesis are activated by curcumin. Curcumin has a notable effect on the Wnt/β-catenin signaling pathway, which is essential for the differentiation and proliferation of stem cells. Curcumin also promotes cell survival, proliferation, and differentiation—all essential processes in neurogenesis—by activating the PI3K/Akt pathway (Limcharoen et al., 2021).

##### 3.4.1.3 Enhancement of neural stem cell proliferation

It has been demonstrated that curcumin stimulates neural stem cells (NSCs), the precursor cells that develop into new neurons, to proliferate and differentiate (Limcharoen et al., 2021). This activity is extremely crucial for sustaining cognitive function and mending brain damage, particularly when we age.

##### 3.4.1.4 Protection against neurodegeneration

Curcumin helps shield already-existing neurons from harm in addition to encouraging neurogenesis. It stops the buildup of tau tangles and amyloid plaques, which are indicative of neurodegenerative illnesses like Alzheimer’s. By reducing the formation of these toxic molecules and mitigating neuronal impairment, curcumin supports brain wellness and helps maintain intellectual function (Tang and Taghibiglou, 2017).

##### 3.4.1.5 Mechanisms in Alzheimer’s and other cognitive disorders

**FIGURE 1:**
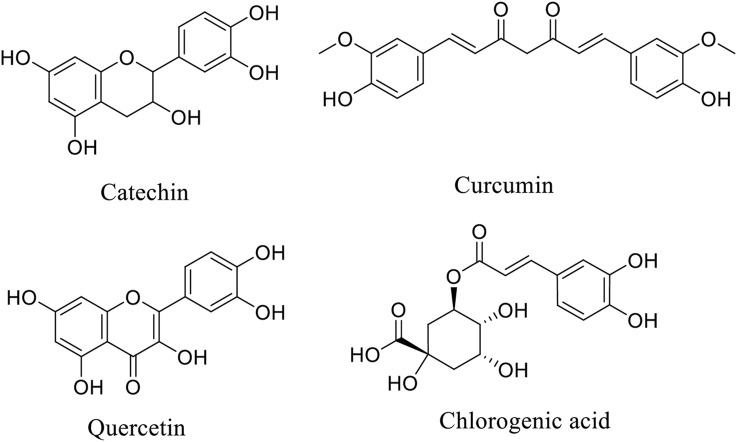
Chemical structure of some important phytonutrients.

Amyloid-beta (Aβ) plaque buildup in the brain, which interferes with normal neuronal activity, is a hallmark of Alzheimer’s disease (Figure 1; Table 1). It has been demonstrated that curcumin binds to amyloid-beta and stops it from clumping together to form plaque. According to certain research, curcumin may even help remove pre-existing plaque by stimulating the brain’s immune cells, known as microglia. Another important element causing neuronal death in Alzheimer’s and other neurological conditions is oxidative stress. As a strong antioxidant, curcumin scavenges free radicals and lessens oxidative damage to proteins, DNA, and lipids. Curcumin supports the integrity of neuronal cells and shields neurons from harm by scavenging these free radicals (Mishra and Palanivelu, 2018). Amyloid precursor protein (APP), the molecule that precedes amyloid-beta, may be processed differently by curcumin. According to research, curcumin may be able to control the activity of molecules that contribute to the formation of amyloid-beta, such as gamma-secretase and beta-secretase (BACE1). Curcumin may aid in lowering amyloid-beta formation by modifying these pathways, potentially reducing the accumulation of amyloid plaques associated with neurodegenerative diseases like Alzheimer’s (Mishra and Palanivelu, 2018).

**TABLE 1:** Phytonutrients and their role to manage the brain health.

Phytonutrient	Source	Impact on brain health
Flavonoids	Berries, citrus fruits, dark chocolate	Antioxidant effects that protect brain cells from oxidative stress, improve cognitive function, and promote neuroplasticity
Carotenoids	Carrots, spinach, kale, tomatoes	Antioxidants that reduce oxidative stress and support cognitive function, particularly in the aging brain
Polyphenols	Green tea, coffee, grapes, berries	Enhance memory, learning, and focus by improving blood flow to the brain and stimulating neurogenesis
Curcumin	Turmeric	Anti-inflammatory and antioxidant effects that protect brain cells from damage and improve mood and cognitive function
Omega-3 Fatty Acids	Flaxseeds, walnuts, fatty fish	Support brain function, improve mood, and reduce inflammation, which can reduce the risk of cognitive decline

## 4 Gut-brain axis and phytonutrients

The gut-brain axis is a model of the reciprocal relationship between the central nervous system (CNS) and the enteric nervous system (ENS) of the gastrointestinal tract, which is connected via sympathetic and parasympathetic nervous system neurotransmitters (Cryan et al., 2019; Holmqvist et al., 2014). Signals can travel from the brain to the GI tract’s muscular, sensory, and secretory capabilities thanks to this two-way communication network. Brain function can be affected by visceral impulses from the GI tract and *vice versa* (O'Mahony et al., 2011). It is currently believed that gut microorganisms play a significant role in the bilateral interaction between the gut and the brain.

The entire population of microbes contributes to equilibrium and carries out vital physiological and metabolic functions for the host (Carabotti et al., 2015; Ma et al., 2019; Rhee et al., 2009). As a result, the gut microbiota is now being explored as a potential target for PD diagnosis and therapy, among other disorders (Riegelman et al., 2024).

Fruits, vegetables, teas, wines, and chocolates are just a few of the foods and drinks that are high in polyphenols, which include both flavonoids and non-flavonoids. Isoflavones, flavones, flavanones, flavanols, and anthocyanins are examples of neuroprotective polyphenols (Ayaz et al., 2019; Koponen et al., 2007).

Foods and drinks high in polyphenols have been associated with a lower incidence of AD, PD, and other neurological diseases. Flavonoids are well-known for their strong anti-inflammatory and antioxidant properties among the several polyphenolic chemicals that make up the class (Brodowska, 2017; Määttä-Riihinen et al., 2004). Because of their capacity to scavenge reactive oxygen species (ROS), traverse the blood-brain barrier, and regulate signaling pathways essential for neuroinflammation, mental processes, and neuronal survival, they have a neuroprotective function (Maleki et al., 2019; Ullah et al., 2020). Flavonoids may also promote the formation of SCFAs with neuroprotective qualities (J. Liu et al., 2020; Vasco et al., 2009). Examples of flavonoids that have a favorable impact on gut microbial populations include quercetin, kaempferol, and catechins. These flavonoids promote the growth of beneficial bacteria while suppressing dangerous ones. By boosting Bacteroidetes and lowering Firmicutes, this approach may help lower inflammation and obesity (Makarewicz et al., 2021; Mithul Aravind et al., 2021).

Terpenoids are a class of lipid-soluble compounds that can be used to treat neurological illnesses because of their antioxidant and anticholinergic qualities (Sharifi-Rad et al., 2022; Simirgiotis et al., 2009).

For instance, preclinical studies have shown that ginkgolides and other specific terpenoids derived from Ginkgo biloba can boost blood flow to the brain, neutralize free radicals, and decrease Aβ neurotoxicity (Nowak et al., 2021). Similarly, terpenoids found in Melissa officinalis and Panax ginseng have antioxidant qualities and have been shown to improve learning and memory (Kennedy, 2019). The cholinesterase-inhibiting properties of phenolic diterpenes, such as rosmarinic acid derived from Rosmarinusofficinalis, may also contribute to enhanced cognitive abilities in AD patients (Määttä-Riihinen et al., 2004).

Alkaloids are naturally occurring nitrogenous compounds with a broad range of biological activity that are present in many plants. Some of these compounds, such as nicotine, berberine from goldenseal, and curcumin from turmeric, have demonstrated potential as neuroprotective and anti-AD agents (Piccialli et al., 2022; Sharifi-Rad et al., 2022; Vasco et al., 2009). These compounds block the enzymes acetylcholinesterase and butyrylcholinesterase while also activating muscarinic receptors, which enhances their anti-neurodegenerative effects as they also function as agonists of dopaminergic and nicotinic cholinergic receptors as well as preventing α-synuclein aggregation (Kim et al., 2024; Simirgiotis et al., 2009).

### 4.1 Gut microbiota’s function in brain health

The two most prevalent bacterial phyla in healthy individuals’ gut microbiota are Firmicutes and *Bacteroides*, which can affect the host’s neurological, immunological, neuroendocrine systems, and metabolic systems (Dinan and Cryan, 2017a; Foster K. R. et al., 2017; Naseribafrouei et al., 2014). The intestinal microbiota may communicate with the brain through the vagal nerve, tryptophan metabolites, and microbial products such as peptidoglycan or short-chain fatty acids (SCFAs) (Dinan and Cryan, 2017b; Foster J. A. et al., 2017). The gut microbiota may impact brain function by affecting glutamatergic, serotoninergic, noradrenergic, dopaminergic, and GABAergic neurotransmission (Fendt et al., 2008; Winter et al., 2018). The microbiota in our gut may affect the production and metabolism of neurotransmitters or even produce them on their own: *Lactobacillus* produces acetylcholine, *Bacillus* and *Serratia* produce dopamine, *Candida*, *Enterococcus*, *Streptococcus*, and *Escherichia* are responsible for serotonin (Dinan et al., 2015), while Bifidobacterium and *Escherichia* produce GABA, and *Escherichia*, Sacchromyces, and *Streptococcus* produce norepinephrine (Dinan et al., 2015; Dinan and Cryan, 2017b).

Except for GABA, which may cross the blood-brain barrier via GABA transporters, there is very little chance that neurotransmitters manufactured by the intestines will make it to the brain. On the other hand, gastrointestinal neurotransmitters may operate on the ENS to indirectly affect brain function (De Caro et al., 2019). Enzymes found in the gut microbiota regulate the processes via which tryptophan is broken down to produce kynurenine, serotonin, or indole. Accordingly, bacteria change the brain’s serotonin levels by influencing the quantity of tryptophan, a precursor to serotonin (Agus et al., 2018).

The human stomach microbiota and phytonutrients have a complex two-way relationship. The gut microbiota is altered by phytonutrient absorption, which suppresses pathogens and encourages the development of good bacteria (Nie et al., 2019). Phytonutrients then influence the synthesis of their metabolites as well which alters the environment in the gut by preventing the synthesis of toxic substances such as hydrogen sulfide, lipopolysaccharide, and indole (Higdon and Frei, 2003). According to Duda-Chodak et al. (2015) and Koutsos et al. (2017), polyphenols found in teas like green and black may help prevent the spread of dangerous germs including *Salmonella typhimurium*, *Helicobacter pylori*, *Listeria* monocytogenes, *Escherichia coli*, *Staphylococcus aureus*, and *pseudomonas aeruginosa*. Meanwhile, the microbiota in our gut produces metabolites such as SCFA and other bioactive compounds. These metabolites stimulate the growth of our gut flora and can improve gut health by targeting numerous processes in the gastrointestinal tract, liver, and pancreas (Huang et al., 2019).

**FIGURE 2:**
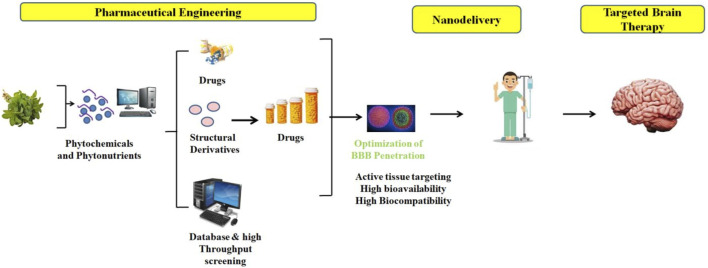
An example of how diverse engineering techniques are being used to phytochemicals to create clinical and commercially viable medications; nano-delivery platforms may be able to maximize their use in the treatment of brain diseases.

Chlogenic acid and similar chemicals can be metabolized by the gut’s indigenous bacteria. Caffeic acid is released during this process, and further metabolism yields derivatives of benzoic, phenyl-propionic, and phenylacetic acids. According to Kim et al. (2014), these phytoconstituents are absorbed into the bloodstream and go to receptors where they bind to provide pharmacological effect (Figure 2).

#### 4.1.1 Gut microbiota and catechin

A wide range of foods, including tea, chocolate, fruit such as apples, and kiwi fruit, contain catechins, which belong to the flavan-3-ol polyphenol family (Zanwar et al., 2014). While polyphenols are prevalent in green tea and its extracts, catechins comprise one-third of the polyphenols in brewed green tea. According to Liu et al. (2018), only a tiny percentage of the catechins that are taken physically are really accessible; the majority passes through the portal vein and into the liver, where phase II enzymes transform them into derivatives of methyl, glucuronide, and sulfate. The gut bacteria play a major role in the biotransformation of catechins producing their metabolites. The colon becomes a bioreactor with a vast capacity to metabolize catechins due to the vast gene pool of gut bacteria (Van Duynhoven et al., 2011). The gut microbiota may use glycosidic bonds, C-ring fission, and other processes to break down the heterocyclic configurations of catechins into smaller compounds such as phenylvalerolactones and phenylvaleric acids (Li and Van de Wiele, 2023). These newly produced microbial compounds may enter the circulation after navigating the colon epithelium (Luca et al., 2020). Microbial biotransformation may produce catechin metabolites with more biological activities than the underlying components (Chen et al., 2020). Catechins may encourage the growth of beneficial gut bacteria, which might result in prebiotic effects, according to *in vitro* research (Hursel and Westerterp-Plantenga, 2013). According to *in vitro* fermentation research, green tea’s catechins boost the numbers of beneficial bacteria like *Lactobacillus* and Bifidobacterium species while lowering those of harmful bacteria like *Clostridium* species. Several animal studies have shown that catechins reduce the ratio of Firmicutes to Bacteroidetes while increasing the diversity of the gut microbiota (Wang et al., 2018a). Recent research suggests that catechins may regulate gut flora to produce therapeutic benefits and effects similar to those of prebiotics. Specifically, after using catechins, the populations of *Bacteroides* and Firmicutes decreased while those of Proteobacteria and Actinobacteria increased in the intestinal mucosa of people with inflammatory bowel disease (IBD) (Barnett et al., 2013).

#### 4.1.2 Gut microbiota and curcumin

The primary curcuminoids found in the rhizomes of plants belonging to the Araceae and Zingiberaceae families are curcumin. Turmeric, a popular Asian spice, uses it as an active component and is used as a culinary colorant, nutritional spice, and herbal medicine (Shen and Ji, 2019). While curcumin metabolism mostly occurs in the liver, the intestines and gut flora additionally contribute significantly to this process. Dihydrocurcumin, tetrahydrocurcumin, hexahydrocurcumin, and octahydrocurcumin (Dei Cas and Ghidoni, 2019; Pandey et al., 2020) are the products of curcumin’s double bond breakdown in hepatocytes and enterocytes. According to earlier studies, bacterial species rose 69% in those receiving curcumin treatment while they fell 15% in the control group. Many Blautia species saw a decline in relative abundance as a result of the treatment, however the following species showed steady increases: *Bacteroides*, *Clostridium*, *Citrobacter*, *Enterobacter*, *Enterococcus*, *Klebsiella*, and *Pseudomonas*. Curcumin dramatically changed the ratio of beneficial to pathogenic microbiota by decreasing the number of Prevotellaceae, Coriobacterales, Enterobacteria, and Enterococci and increasing the number of butyrate-producing bacteria, Bifidobacteria, and Lactobacilli. Changes to the intestinal microbiota may also help clarify the way curcumin decreases hyperlipidemia and controls immunological responses, in addition to its anti-inflammatory properties and anti-colonotropic carcinogenicity characteristics (Peterson et al., 2018).

#### 4.1.3 Gut microbiota and quercetin

Red wine, apples, kale, onions, and cherries are all rich sources of quercetin, a polyphenolic flavonoid. When quercetin is attached to sugar moieties like rhamnose or rutose, it produces quercetin glycosides and rutin by replacing one of the OH groups with a sugar group (Guo and Bruno, 2015). The liver is where quercetin is mostly processed. Quercetin is transported to the liver for metabolism after absorption. Here, it passes through phases I and II, producing metabolites that are then transported by the circulation to various bodily locations (Dabeek and Marra, 2019). Quercetin’s environmental effects on the gut are significant enough to affect the regulation of the microbiota.

Food-pathogenic bacteria including *Vibrio* parahaemolyticus, *E. coli*, *S. aureus*, and *Listeria* monocytogenes may be present in the human gut microbiota. Infections linked to hospitals or the community may also be clinically significant. Quercetin’s antibacterial and prebiotic properties may help reduce the number of harmful microbes in the stomach (Lan et al., 2021; Liu et al., 2019; Xie et al., 2017). The study by Lan et al. found that quercetin administration increased gut microbiota diversity and resulted in noticeable alterations in the three main groupings of gut microbiota (Bacteroidia, *Clostridium*, and Bacilli). Ruminococcus decreased and *Lactobacillus* increased after quercetin administration (Lan et al., 2021).

#### 4.1.4 Gut microbiota and chlorogenic acid

Chlogenic acid (CGA), one of the most prevalent polyphenols in the human diet, is found in a variety of foods and plants, including tea, coffee, wormwood, apples, and coffee seeds (Naveed et al., 2018; Nwafor et al., 2022). It provides several health benefits. Because of its anti-oxidative, anti-inflammatory, anti-cancer, and anti-neurodegenerative properties, chlorogenic acid has shown a number of positive benefits (Naveed et al., 2018). Because of its hydrophilic nature, chlorogenic acid has poor oral bioavailability and absorption due to its inability to cross lipophilic membrane barriers (Cowan et al., 2014). While the small intestine absorbs a little amount of chlorogenic acid, the gut bacteria break it down into smaller pieces in the large intestine (Chen et al., 2018; 2019). Rapid hydrolysis of chlorogenic acid by resident microflora in the colon and subsequent further metabolism by host enzymes might lead to the release of other metabolites into the circulatory systems. Researchers discovered that the coffee’s chlorogenic acids were rapidly broken down by the colonic microbiota, resulting in the production of eleven catabolites after just 6 h of incubation. By regulating the populations of some advantageous microorganisms (such as Burkholderiales, Desulfovibrio, *Klebsiella*, Desulfovibrionales, and Bifidobacterium), chlorogenic acid may benefit the host (Song et al., 2019).

### 4.2 How phytonutrients support a healthy gut brain axis

A variety of neurological disorders, including PD, MS, depression, anxiety, and ASD, have been shown to begin and progress in part because of GBA (Cryan et al., 2020). Crucial to the GBA is the gut microbiota, which consists of resident bacteria of the gastrointestinal tract and may influence behavior and brain function via many routes (Oslovsky et al., 2020). Modifying the gut microbiota may have therapeutic advantages for a number of ailments. Numerous neurological conditions have been connected to dysbiosis, a disruption in the makeup of the gut microbiota (Moss et al., 2018). Phytochemicals may be useful in the treatment and prevention of neurological illnesses because to their neuroprotective, anti-inflammatory, and antioxidant qualities as well as their ability to alter the gut microbiota (Luo et al., 2021). Some evidence suggests that curcumin, a polyphenol found in turmeric, may be useful in the treatment with multiple sclerosis due to its anti-inflammatory, antimicrobial, and gut microbiota-modulating properties (Hewlings and Kalman, 2017). Red wine and grapes are potential sources of the polyphenol resveratrol. Research suggests it may be able to alter the gut microbiota and exert neuroprotective effects, which could lead to a cure for PD (Rana et al., 2022; Wang et al., 2018b). Numerous fruits and vegetables contain quercetin, a flavonoid with anti-inflammatory, antioxidant, and gut flora-modifying qualities. These properties could be the cause of quercetin’s antidepressant effects (Kawabata et al., 2015). Microbiota enzymes in the stomach may change polyphenols by stripping them of sugar molecules, adding hydroxyl groups, and stripping them of methyl groups. This makes the breakdown products smaller and easier for the intestines to absorb (Keppler and Humpf, 2005; Makarewicz et al., 2021). Some of the byproducts of these reactions retain biological activity at a greater level than the parent chemical, while others completely lose it. This provides further evidence that treating severe neurological illnesses by targeting the GBA might be a viable strategy (Jaberi et al., 2024).

## 5 Clinical studies and emerging evidence

### 5.1 Overview of human and animal studies on phytonutrients in brain health

The fact that phytonutrients affect synapse and neuronal activities is supported by several lines of data. It is well known that they improve cognitive functioning by modulating CREB signaling networks and the kinase extracellular signal-regulated (ERK) and by protecting against various types of damage brought on by reactive oxygen species (ROS), such as lipid peroxidation and neuroinflammation (Spencer, 2009). The neuroprotective effects of polyphenols may be due to their involvement with signaling pathways that keep energy levels stable or their ability to protect neurons from oxidative damage, among other possible mechanisms. Table 2 displays some of the most current findings from animal models about the capacity of polyphenols to enhance cognition. Additionally, Table 3 summarizes a number of clinical studies (Mohandas et al., 2014).

**TABLE 2:** Neuroprotective phytochemicals in animal models of brain injury.

Phytochemical	Animal model	Mechanism of action	References
Curcumin	Male Wistar rats	Enhanced cognitive function and reduced oxidative damage caused by phenytoin	Reeta et al. (2009)
Luteolin	Rat	Reversed the chronic cerebral hypoperfusion-induced learning and memory loss caused by cAMP response element binding protein (CREB) activation	Xu et al. (2010)
Resveratrol	Rats	BDNF activation and antidepressant effect	Hurley et al. (2014)
Extracts of *Bacopamonnieri*	300–450 mg for human adults free of dementia	Improvements in brain function	Pase et al. (2012)
Epigallocatechin-3-gallate (EGCG)	Rats	Reduced cognitive impairment brought on by pentylenetetrazole (PTZ)	T. Xie et al. (2012)
*Cantellaasiatica* water extract	Tg2576 mice	When taken orally, it protected against oxidative damage, reduced anticholinergic activity, and reduced behavioural impairments associated with β-amyloid	Soumyanath et al. (2012)
Ethyl acetate extract of fruits of*Morindacitrifolia*Linn.	Mice	Improvements in memory (both short and long term), curiosity, and the ability to absorb new information are all results of an antioxidant enzyme system that includes a rise in dopamine and serotonin levels and a decrease in MAO and AChE activity	Muralidharan et al. (2010)
Rutin and Quercetin	Zebra fish	Enhanced antioxidant mechanisms and reduced oxidative stress Enhanced cholinergic system function and protected against scopolamine-induced amnesia	Richetti et al. (2011)
α-asarone	Mice	Enhancement of memory and cognitive function by modulation of the antioxidant defense system and suppression of AChE activity	Kumar et al. (2012)
Saffron	Mice of both young and old age	Enhancement of antioxidant mechanisms and amelioration of oxidative stress lead to better learning and memory	Papandreou et al. (2011)
Dried ginger extract	Mice	Reduced impairments in learning and memory pathways generated by scopolamine–induced stimulation of ERKCREB signaling and hippocampal synaptogenesis by NGF	Lim et al. (2014)
Blackberry	Old rats of 19 months	Enhanced antioxidant and anti-inflammatory response; enhanced cognitive and motor abilities	Shukitt-Hale et al. (2009)
Citrus 5-hydroxy-3,6,7,8,3,4-hexamethoxyflavone	PC12 pheochromocytoma cells	The cAMP/PKA/CREB Pathway for the Promotion and Modulation of Neurite Outgrowth	Lai et al. (2017)
Extract of *Cinnamomumzeylanicum*	Rats	Reduced oxidative stress indicators and recovered amnesia caused by scopolamine	Jain et al. (2015)
Tenuifolin extracted from *Radix polygalae*	Aged and amnesic mice	Enhancement of learning and memory by lowering AChE activity in the cortex and raising NE and DA levels in the hippocampus	Zhang et al. (2008)
Yam from *Dioscorea pseudojaponica Yamamoto*	Mice	Memory and learning on the Morris water maze test are both improved by increasing the body’s natural antioxidant enzymes	Chiu et al. (2009)

**TABLE 3:** Few clinical studies of plant extracts in cognitive enhancements.

Phytochemicals	Animal model	Mechanism of action	References
Extract of *Centellaasiatica*	28 healthy elderly participants	Reduce the deterioration of cognitive abilities and mood disorders that occurs naturally with aging in healthy older adults	Wattanathorn et al. (2008)
Extract of *Melissa officinalis*	Patients with AD (between 65 and 80 years old)	Much more effective than a placebo in enhancing cognitive function	Akhondzadeh et al. (2005)
Extract of *Bacopamonnieri*	72 healthy urban adults	Enhanced mental performance and decreased anxiety levels Memory acquisition and retention are markedly enhanced in healthy elderly Australians	Downey et al. (2013), Morgan and Stevens (2010), Sathyanarayanan et al. (2013)
Caffeine		Enhance customers’ mental state and boost their productivity	Haskell et al. (2005)
Extract of *Salvia lavandulaefolia*		Acute mood and cognitive change in young healthy individuals	Tildesley et al. (2005)
Monoterpenoid extract of sage (*Salvia lavandulaefolia)*		Benefits of cholinergic modulation include less mental tiredness and enhanced alertness	Kennedy et al. (2011)
Saffron from *Crocus sativus*	40 adults who were found to meet the criteria for a mental disorder according to the Diagnostic and Statistical Manual of Mental Disorders	Much superior results compared to the placebo on the Hamilton depression rating scale	Akhondzadeh et al. (2005)
Nicotine		Muscle memory, attention (both alert and oriented), and working memory (both episodic and continuous) are all improved	Heishman et al. (2010)
Caffeine	21 prepubescent youngsters, 8–12 years old (9 of them were female) Experimental design: Double-blind, placebo-controlled, crossover trial (1 week of treatment)	While caffeine may improve performance on attention and motor task tests, it also has the potential to raise anxiety	Bernstein et al. (1994)
	12 studies, 23 datasets and 346,913 individuals Experimental design: review and meta-analysis of observational study on depression	To a lesser extent than with tea and caffeine, coffee protects against depression	Grosso et al. (2016)
	59 people (15 females) divided into two age groups: those between the ages of 20 and 34 and those between the ages of 61 and 80. Experimental design: Randomized, double blind, placebo controlled, counter balanced cross over trial	Further study into the psychoactive effects of coffee is needed, since these results indicate that coffee has behavioral effects beyond its caffeine concentration. This raises concerns about using decaffeinated coffee as a placebo	Haskell-Ramsay et al. (2018)
Flavanoids	24 men aged between 30 and 65 years Experimental design: Randomized, double blind, placebo controlled trial	Following consumption of the flavonoid-rich beverage, there was a significant improvement in executive function and psychomotor speed as compared to the control group	Alharbi et al. (2016)
	18 healthy men (average age 23 years) Experimental design: Randomized, double blind, placebo controlled cross over trial	Improve blood oxygenation and cognitive performance	Gratton et al. (2020)
Phenolic acids (chlorogenic acid (CGA)	38 healthy participants between 50 and 69 years (17 female) reported problems with their subjective memory Experimental design: Randomized, double blind, placebo controlled, parallel trial	CGAs have the potential to enhance many cognitive processes, including attention and motor speed, which might lead to more effective completion of challenging tasks. It is possible that the enhanced cognitive abilities seen in the neuropsychological tests are reflected in the higher blood concentrations of TTR and ApoA1 after CGA therapy	Saitou et al. (2018)
Anthocyanins	26 healthy elderly with an average of 68.3 ± 1.7 years, 13 of whom are female Experimental design: Randomized, double blind, controlled trial	In healthy older people, supplementing with a blueberry concentrate high in anthocyanins increased blood flow to the brain and activity in regions linked to cognition	Bowtell et al. (2017)
Carotenoids	2,983 adults aged between 45 and 60 years, 1,381 of them being female Experimental design: Randomized, double blind, placebo controlled, primary prevention trial	These results, might suggest that a diet rich in a variety of fruits and vegetables, especially those with vibrant colors, may aid in the preservation of brain function as we age	Kesse-Guyot et al. (2014)
	49 healthy non-smoking women of aged between 60 and 80 years Experimental design: Randomized, double blind, intervention trial	According to these exploratory studies, supplementing with carotenoids like DHA and lutein may provide cognitive advantages for older persons	Johnson et al. (2008)
Flavonoids and anthocyanins	25 healthy mothers of age of 43 years Experimental design: Randomized, double blind, placebo controlled cross over trial	The cognitive benefits of drinking grape juice, which are rich in flavonoids, are not limited to people with mild cognitive impairment; they are noticeable even when doing simple everyday tasks like driving. Additionally, the effects may last even after stop drinking grape juice	Lamport et al. (2016)
Flavonoids	90 elderly person (53 female) of age between 61 and 85 years. Experimental design: randomized, double-blind, controlled, parallel-arm	Some indicators of cognitive decline with age may be mitigated by a diet rich in cocoa flavanols, which may work via increasing insulin sensitivity. The results of this study provide further evidence that a diet rich in flavanols may help maintain cognitive function as we become older	Mastroiacovo et al. (2015)
Anthocyanins	14 children (4 female) of 9 years age Experimental design: Controlled and cross over trial	The results of this pilot trial were mixed, but the enhancements in delayed recall seen in the control group indicated that school-aged children encoded memory objects more successfully after acute flavonoid-rich blueberry treatments	Whyte and Williams (2015)
	31 individuals (9 female) with mild cognitive impairment of aged 73 years Experimental design: Randomized, double blind, placebo-controlled trial	Older persons with mild cognitive impairment had their TNF-α concentrations decreased after 8 weeks of taking a high dosage of fruit-based anthocyanins daily. There was no change in microvascular function, blood pressure, or other inflammatory indicators when anthocyanins were added	Do Rosario et al. (2021)
	111 healthy elderly person (58 female) of aged between 60 and 72 years Experimental design: Randomized, double blind, placebo-controlled trial	The findings demonstrate that healthy older persons may safely supplement with Cognigrape® for 12 weeks, which can enhance physiological cognitive profiles and simultaneously relieve unfavorable neuropsychological status	Calapai et al. (2017)
Phenolic acids	142 healthy individuals (44 female) of aged between 18 and 50 years Experimental design: Randomized, double blind, placebo-controlled, parallel trial (90 days)	This research adds to the growing body of evidence that PSE is an effective nootropic and enhanced cognitive functions, as it shows that a young, active population may benefit from taking 900 mg of PSE daily	Falcone et al. (2019)

### 5.2 Current research gaps

Researchers are anticipating that plants containing many bioactives may hold the key to understanding cognitive diseases, which are notoriously complicated, by acting on ligands directed to several targets simultaneously. Phytomedicines have a lower potential for adverse effects such as nausea, vomiting, dizziness, diarrhea, and seizures compared to traditional cognitive-enhancing medications. Having said that, addiction may be caused by certain phytonutrients like cocaine (Steiner and Van Waes, 2013). Thorough research on their effectiveness, toxicity, and safety is necessary (Mehta et al., 2012). Given the promising therapeutic potential of cognitive enhancers generated from natural sources, there is an urgent need to provide extensive evidence on their safety, effectiveness, and toxicity (Mohandas et al., 2014).

## 6 Dietary sources and recommendations

### 6.1 Phytonutrients

**FIGURE 3:**
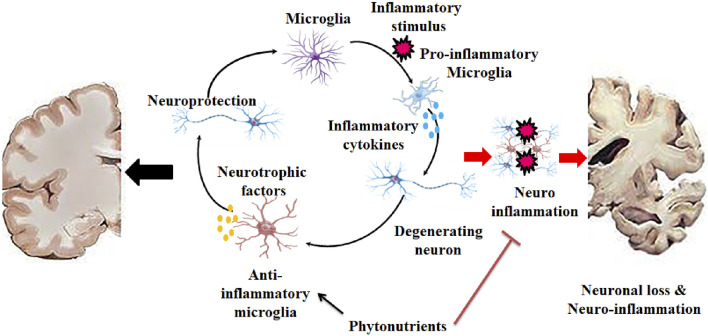
Initiation of neurodegeneration by triggering neuroinflammation where microglia paly key role in both neuroprotection and neuronal loss.

**FIGURE 4:**
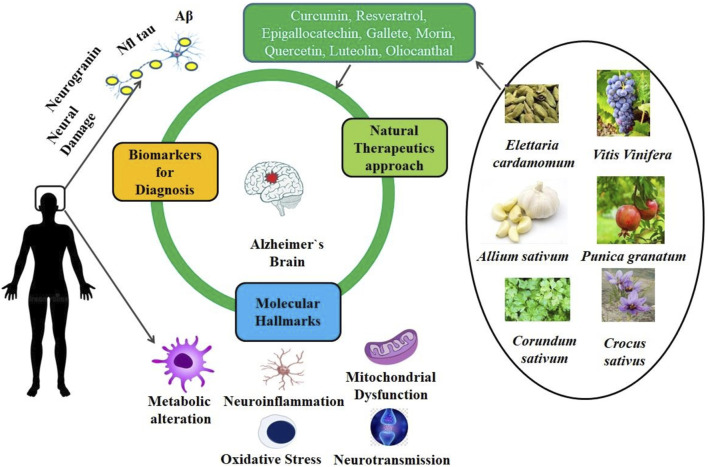
Therapeutic effects of phytonutrients and secondary metabolites to reduce neuroinflammatory condition in Alzheimer's disease.

Dietary plants have been shown to have over 10,000 phytonutrients (Jeffery et al., 2003; King and Young, 1999). Species and cultivars range significantly in their concentrations, as do agricultural practices (such as fertilization and irrigation), storage, processing, and domestic use (Amiot et al., 2012). Figures 3, 4 depict the major families and molecular makeup of phytonutrients contained in dietary plants. Because of their characteristics, phytonutrients can influence metabolic syndrome and related processes, including oxidation and inflammation (Amiot et al., 2016). According to experimental research conducted on cells or animals, their mechanisms of action are anti-inflammatory, anti-cancer, anti-microbial, and antioxidant, suggesting their potential therapeutic value in the prevention and treatment of various chronic diseases (Zhang and Tsao, 2016; Khameneh et al., 2019; Sonawane et al., 2016; Xiao, 2017; Kaulmann and Bohn, 2014) (Figure 3).

#### 6.1.1 Phenolic acids

One of the most prevalent plant non-flavonoid phenolic substances is phenolic acid, also known as phenolcarboxylic acid, which is a member of the polyphenol family (Dinkova-Kostova and Kostov, 2012). They include one phenolic hydroxyl and at least one carboxylic activity (Liu, 2004). Cinnamic acid and its derivatives (ferulic, paracoumaric, caffeic, and sinapic acids) and hydroxybenzoic acid and its derivatives (gallic acid, vanillic acid, parahydroxybenzoic acid, syringic acid, and protocatechic acid) are included in this group (Liu, 2013). Citrus fruits, berries, apples, coffee, kiwis, cereals, wheat flours, onions, and artichokes are just a few of the foods that contain phenolic acids (King and Young, 1999; Kumar and Goel, 2019). Phenolic acids can be produced by the gut bacteria by secondary metabolism of other polyphenols, in addition to dietary sources (Chandrasekara, 2019).

#### 6.1.2 Flavonoids

Isoflavonoids, flavonols, flavones, flavanols, and flavanones are all members of the polyphenol family (Liu et al., 2019; Bruneton, 2016). Although they belong to the flavonoid family as well, anthocyanins are covered in a different paragraph because of their unique characteristics and potential health benefits. Two aromatic rings connected by the three carbons, or C6-C3-C6, make up the majority of flavonoids. Banjarnahor and Artanti (2015), Häkkinen and Törrönen (2000) state that this chain is frequently closed in a C-ring, an oxygenated heterocycle. They are categorized as flavonols, flavones, flavanols, flavanones, anthocyanidins, or isoflavonoids based on variations in the general structure of the heterocyclic C-ring (Banjarnahor and Artanti, 2015; Häkkinen and Törrönen, 2000). Many plants have “colorful hues” due to flavonoids, which are universal pigments that come in yellow, red, and purple colors. Through their function as co-pigments, flavonoids aid in pigmentation even when compounds are not readily apparent. Here, anthocyanosides are protected and co-pigmented by colorless flavones and flavonols. The two main constituents of the flavonoid class known as flavonols, which are some of the most prevalent flavonoids in diet, are quercetin and kaempferol (King and Young, 1999). Numerous plants may contain flavonoids, although only in trace amounts (King and Young, 1999; Bruneton, 2016; Bobinaite et al., 2012). While flavonoids may be found in many different colored plants, they are often found in tea, onions, and apples (Gupta and Prakash, 2014). Citrus fruits, herbs, and tomatoes all contain flavanones. Lettuce, cabbage, onions, and olives all contain flavanols. Olives and celery both contain flavones. Flavanols are found in tea, red wine, and pears. Lastly, soy products are the primary source of isoflavones (Gupta and Prakash, 2014; Amiot et al., 2012; Parker et al., 2007).

#### 6.1.3 Anthocyanins

The general metabolism of flavonoids results in the production of anthocyanins, a subfamily of flavonoids (Liu, 2004; Bruneton, 2016). Red, pink, blue, or purple fruits and vegetables are the most prevalent sources of anthocyanins, including cyanidin, pelargonidin, delphinidin, and malvidin (Lin et al., 2017). Anthocyanins range in hue from vivid orange to purple. The absorbance wavelength changes from orange-colored pelargonidin to purple-colored delphinidin as the degree of hydroxylation increases. Plums, cherries, and berries (including elderberries, blackcurrants, and blueberries) are especially rich in anthocyanins. They may also be found in beverages like red wines and fruit juices, as well as in root vegetables like radish plants, beets, and red onion bulbs (Amiot et al., 2012; Derbel and Ghedira, 2005; Scalbert and Williamson, 2000; Vlachojannis et al., 2015). Anthocyanins are also found in red cabbage and eggplant.

#### 6.1.4 Tannins

Flavonoids and tannins are members of the same phenolic chemical family. Condensed tannins and hydrolyzable tannins are the two categories into which they are separated based on their architecture and biogenetic origin (Ghosh, 2015; Bruneton, 2016; Scalbert and Williamson, 2000). Condensed tannins are chemical substances that are oligomers or polymers made up of units of flavan-3-ols connected by type C4→C8 and/or C4→C6 bonds. They are also referred to as catechins or proanthocyanidins. Although tannins cannot be hydrolyzed, they can break down into colorful pigments called anthocyanidins when heated and handled with an acid (Bruneton, 2016; Scalbert and Williamson, 2000; Chung et al., 1998). After hydrolysis, hydrolyzable tannins, as opposed to condensed tannins, can pass through the intestinal barrier (Amiot., 2012). Pomegranate bark, sorghum, barley seeds, tea, wine, cocoa beans, carob beans, and plums can all contain high levels of tannins (Amiot et al., 2012; Derbel and Ghedira, 2005; Scalbert and Williamson, 2000; Chung et al., 1998).

#### 6.1.5 Organosulfur compounds

Numerous molecular kinds with a similar fundamental chemical structure are included in the class of organosulfur substances (Liu, 2013). Depending on the subclass, an aglycone is made up of an amino acid, the molecular framework of glucose via a sulfur link, and a sulfate band via the nitrogen atom of the methoxy group located around a carbon atom (Sugiyama and Hirai, 2019). Sulforaphanes, isothiocyanates, native compounds, and compounds made from allyl sulfides are all members of the organosulfur biological family (Bruneton, 2016; Amiot et al., 2012). When glucosesinolates hydrolyze, physiologically reactive isothiocyanates are produced (Sugiyama and Hirai, 2019). Glucosinolates and garlic sulfur derivatives are the two organosulfur compounds most frequently present in plant-based diets (Amiot et al., 2012). Particularly in Brassicaceae or cruciferous vegetables such as (cabbage, cauliflower, turnip, broccoli, black radish, and mustard), the content of glucosinolates varies according to the species, plant part, culture, and environment (Derbel and Ghedira, 2005). These substances give forth powerful flavors and aromas. Mustard seeds contain isothiocyanate, although cruciferous vegetables like cauliflower and cabbage are the primary source of sulforaphane (Jeffery et al., 2003). Another excellent source of sulfur compounds is garlic.

#### 6.1.6 Carotenoids

**FIGURE 5:**
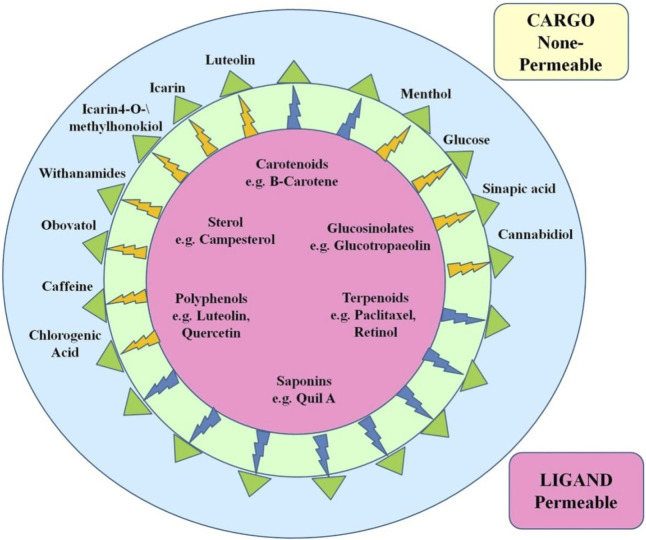
Examples of existing or perhaps useful nanoparticles for in vivo brain therapy applications that are loaded with therapeutically active phytochemicals (inaccessible to the BBB) or embellished with BBB-permeable phytochemicals. Phytochemicals that are bolded have been documented in carlier research using the PubMed database.

Only around 20 of the 800+ distinct molecules that make up the vast family of carotenoids—which range in hue from yellow-orange to red—are found in the diet, according to Amiot et al. (2012) and Bruneton (2016). The general structure of a carotenoid is a polyene hydrocarbon molecule with nine to eleven double-bonded bonds which may end in rings (Fiedor and Burda, 2014). The two types of fat-soluble substances known as carotenoids are carotenes and xanthophylls. Taxaxanthin, zeaxanthin, lutein, and cryptoxanthin are among the compounds of the first class. Lycopene, carotene, and carotene are the symbols for carotenes. Lutein, zeaxanthin, lycopene, and carotene are the antioxidants known as carotenoids that have been the subject of the greatest research. Carotene is the most well-known precursor of vitamin A (Gupta and Prakash, 2014; Fiedor and Burda, 2014). The natural environment is full with carotenoids, which are very vulnerable to oxidation. They are accumulated by the chloroplasts of all photosynthetic tissues (Van der Sluis et al., 2001). Carotene, lutein, violaxanthin, and neoxanthin are found in the leaves of nearly all plants. Fruits can also include carotenoids, such as chloroplastic carotenoids found in flower petals (including those of the common marigold, pansy, and French marigold) and other derivatives (such lycopene and capsanthin). Sweet potatoes, Brussels sprouts, broccoli, kale, carrots, spinach, tomatoes, peppers, citrus fruits, seeds, certain kinds of mushrooms, and leafy greens like lettuce and arugula are among the many plants that contain carotenoids (Derbel and Ghedira, 2005; Bohn, 2019; Niroula et al., 2019; Tan and Norhaizan, 2019) (Figures 4, 5).

#### 6.1.7 Caffeine

1,3,7-trimethylxanthine is another name for caffeine, a member of the alkaloid family (Bruneton, 2016; Ashihara et al., 2008). However, due to its substantial contribution to daily phytochemical intake, demonstrated health benefits, and frequent mention in nutritional guidelines like those published by the French Agency for Food, Environmental and Professional Health and Safety (ANSES), caffeine was added as a family in its own right (Heckman et al., 2010). Coffee, kola nuts, tea, mate leaves, and guarana seeds are all sources of caffeine, the most widely used psychoactive ingredient in the world (Heckman et al., 2010; WHO, 2013).

### 6.2 Recommended intake for different age groups and conditions

The research population consisted of people who participated in the World Health Survey (WHS), a cross-sectional survey conducted in 70 countries between 2002 and 2004 (WHO, 2002; Ustun et al., 2003). Using multistage cluster sampling, the WHS selected a nationally representative group of people who were 18 years of age or older (Singh et al., 2021). Based on location, sex, socioeconomic class, and perhaps one or two other divisions from each participating country, the sample was evenly split across three to five strata (Khan et al., 2022). Sociodemographic data, health status descriptions and evaluations, indicators of risk, chronic illnesses, mortality rates, healthcare utilization, health system responsiveness, and social capital attributes were among the individual-level data gathered by the WHS (Li et al., 2023). The present study focused on male and female WHS members who were 18 years of age or older and answered a survey on the fruits and vegetables they were consuming (Patel et al., 2024c). People in a selection of participating countries (n = 198,637) were given the survey. Reports indicated that daily consumption of fruits and vegetables varied from zero to one hundred servings. The study sample was restricted to individuals who reported eating 15 or less servings of fruits and vegetables per day, a quantity equal to the 99th percentile of adult consumption when age, sex, and fruit and vegetable serving statistics are included. This was done in order to eliminate 1,712 participants. The total adult population sampled consisted of 196,925 individuals. Interviewers obtained participants’ written agreement prior to the interview, and participation in the study was entirely voluntary (Food and Agricultural Organization of the United Nations: FAO, 2013; Ustun et al., 2003).

#### 6.2.1 Levels of total fruit and vegetable consumption in the study population

Participants in the World Health Survey (WHS) came from 52 geographically diverse nations, primarily low- and middle-income countries, and were administered the extended version of the questionnaire, which included specific questions regarding their fruit and vegetable consumption (FAO, 2013; Patel et al., 2022; Beking and Vieira., 2011). They were asked two primary questions: 1 How many servings of fruit do you usually consume in a day? and 2 How many servings of vegetables do you typically consume each day? To simplify reporting and avoid the need to average intake over multiple days, participants were instructed to consider a “typical day” as any day when they consumed fruits or vegetables (FAO, 2013; Khan and Lee, 2023). Trained interviewers showed participants visual aids—cards illustrating standard serving sizes of fruits and vegetables commonly consumed in their region (Zhao et al., 2024). Based on the survey guidelines, a standard serving of vegetables was generally considered to be one cup of raw leafy greens (like spinach or salad), half a cup of cooked or raw chopped vegetables (such as carrots, tomatoes, pumpkin, Chinese cabbage, maize, or beans), or half a cup of vegetable juice (Wang et al., 2025). For fruits, a single serving was defined as one medium-sized fruit (such as a banana, apple, or orange), half a cup of chopped, cooked, or canned fruit, or half a cup of fruit juice (Chen et al., 2021). Because the questionnaire did not collect data on the regular consumption of other food groups, this study focused exclusively on phytonutrient intake derived from fruits and vegetables (Patel et al., 2022).

#### 6.2.2 Fruits and vegetables available by geographical region

The WHO Global Environment Monitoring System/Food Contamination Monitoring and Assessment Programme (GEMS/Food) developed a methodology for grouping countries with similar dietary consumption into clusters and establishing a representative diet for each of these clusters starting in the early 1990s. The WHO and FAO provided quantitative data that demonstrated the availability of specific fruit and vegetable kinds by nation, but the WHS did not record the exact types of fruits and vegetables that each respondent consumed. The *per capita* food availability information that serves as the foundation for the diets is derived from the FAO’s annual statistics on farming, production of food, imports, and exports, which are released as FAO supply utilization accounts (Sy et al., 2013). The amounts of food that are produced plus imported, adjusted for exports, and used for seed or animal feed are all included in the statistics on food availability, which displays the total amount of food available per person. These figures serve as a proxy for real consumption rather than reflecting it. Based on FAO data from 1997 to 2001 and the geographic proximity of the countries within the statistical clusters, the WHO designated geographic diet groupings, known as GEMS/Food clusters, using a letter-coding scheme (A to M) in 2006 and used cluster analysis to divide the world’s countries into thirteen statistical clusters of seven to twenty-two counties each (FAO, 2013). Geographic proximity was not taken into consideration when the WHO created new cluster groups and diets in 2012 based on FAO data from 2002 to 2007. We produced updated food consumption estimations for the thirteen regions identified by the 2006 regional diet clusters using this data on food availability. This involved weighting the data for each country according to its population size and calculating weighted average consumption quantities for each regional diet cluster. The FAO numbers were changed from kg/year to g/d for the purposes of this study. The FAO data from 2002 to 2007 were organized in a hierarchical framework of 18 major food categories, and estimates of food accessibility were supplied for 415 different food categories or combinations of related foods. Sixty-seven fruit types and forty-two vegetable categories were chosen for this study. Olives and their liquids were categorized as fruit, but nuts and seeds were not. Pulses, herbal cures, roots, and tubers like potatoes and cassava were not included in the vegetable categories, but juices were. “Not elsewhere specified,” or “nes,” was a description used in some of the FAO-approved food categories. The FAO utilized the “nes” categories to record the availability of foods that did not fit into a certain category. Some countries probably utilized the “nes” categories to report on foods that were already under a specific category, especially if such foods were not very important in their local communities (Sy et al., 2013; ARS, 2012). Through each geographic diet cluster, food intakes compared to fruits and vegetables labeled as “nes,” except for the “Juice of Vegetables nes” and “Fruit Prpnes” categories, were distributed to the appropriate fruits and vegetables groupings in proportions that reflected the corresponding abundance of nourishment in each category.

For instance, the consumption of “Pome Fruit Nes” was divided across the distinct categories of “Apples,” “Pears,” and “Quinces” based on the total consumption of the fruit as well as the relative availability of each of these three categories across each regional diet cluster. This methodology is in line with earlier evaluations of food consumption based on FAO data (A Vieira, personal communication) (FAO, 2012). The previously assigned “nes” categories were reallocated to create 48 vegetable categories and 58 fruit categories.

#### 6.2.3 Phytonutrient concentration data for categories of fruits and vegetables

Data on phytonutrient concentrations for certain antioxidants, flavonoids, and a phenolic acid (ellagic acid) were available for specific fruits and vegetables in each of the FAO vegetable and fruit categories. This study includes nine phytonutrients, which are primarily present in vegetables and fruits and belong to the major classes of phytochemicals. We determined how much was consumed of a hormone called quercetin for the flavanone and flavonol subclasses of flavonoids, respectively. These two flavonoids comprise the majority of the flavonoid subclasses ingested in the diet, according to previous studies conducted with a Spanish population (Sy et al., 2013). The National Nutrient Database for Standard Reference, which contains Release 25 of the United States Department of Agriculture (USDA), was used to determine the amounts of carotenoid pigments (a-, b-, b-cryptoxanthin, lutein/zeaxanthin, and lycopene) (Bhagwat et al., 2013; Cuartero et al., 2011). Both public and unpublished sources provided information for this database. Carotenoid levels for two relevant food categories—cassava leaves and cashew apples—were absent from the USDA database. The b-Carotene values for these items were obtained from another data source (Crozier et al., 1997; Daniel et al., 1989). The values for hesperetin, quercetin (measured as mg aglycone/100 g edible part), and anthocyanidins (the total of cyanidin, delphinidin, malvidin, pelargonidin, peonidin, and petunidin) were obtained using the most recent edition of the USDA’s flavonoid database (Ali et al., 2011). Methodically gathered from several foreign sources, the USDA database includes analytically comprehensive flavonoid data. The single or most important source of flavonoid concentration-related information utilized in several international studies on phytonutrient intakes has been this database of flavonoid values. With moisture content adjustments, the flavonoid levels for concentrated juices and food that had dried that were lacking were filled in using the information from single-strength juices or food in its natural form. Flavonoid levels for missing cooked meals were computed using raw food data, assuming a 25% retention rate (Amakura et al., 2000). There is no information on ellagic acid concentrations in the USDA databases. The ellagic acid equivalent amount of foods classified as FAO fruits and vegetables was derived using a database of values collected from published studies (Anttonen and Karjalainen, 2005; Williner et al., 2003). The database of ellagic acid values contained ellagic acid from a range of sources (such as free ellagic acid, ellagitannins, and others), with the findings provided as ellagic acid equivalents. The tests were carried out after acid hydrolysis. For seventeen different fruit varieties that were part of the study, ellagic acid amounts that were not zero were discovered. A number of the FAO’s fruit and vegetable classes corresponded to particular goods, such spinach or bananas. Some categories, such as “Carrots and turnips” and “Cabbages and other brassicas,” however, had many meals. The available phytonutrient data pertaining to the typical raw and/or cooked forms of the food(s) that would be typically ingested were matched with each FAO category. Based on averages of the data on phytonutrient content for turnip greens, mustard greens, radish, kohlrabi, collards, cabbages, Brussels sprouts, and cress, the category “Cabbages and other brassicas” was developed for this study (Bowman et al., 2013; Cuartero et al., 2011). Because there was a lack of more specific information on the consumption of specific foods within these broad categories, the available phytonutrient concentration measurements were averaged to create a representative phytonutrient concentration in order of the profile (phytonutrient amount per 100 g of food as consumed) for each FAO category. Among the fruits or vegetables that belonged to certain groups, the observed anthocyanidin quantities differed significantly. Red grapes, for instance, which are categorized as “Grapes,” contain a relatively high concentration of anthocyanidins (Ali et al., 2011; Daniel et al., 1989). However, the USDA flavonoid database does not include information on the anthocyanidin concentrations of green grapes, which are also categorized as “Grapes,” despite the fact that they are likely not a substantial source of these flavonoids (Cantos et al., 2002). For items (such green grapes) with lacking anthocyanidin data, we assumed a zero value to give a more realistic average anthocyanidin concentration. What is considered inadequate consumption of fruits and vegetables? The WHO panel on food, nutrition, and the prevention of chronic illness recommended 400 g or more of fruits and vegetables per day, excluding potatoes, cassava, and other tubers (WHO/FAO, 2003). 400 g of fruits and vegetables, or at least five servings daily with an average serving size of 80 g, is considered poor intake according to WHO standards (Hall et al., 2009; Table 4).

**TABLE 4:** Summary of major phytonutrients and their neuroprotective effects.

Phytonutrient	Natural sources	Associated brain disorders	Proposed mechanisms of action	Supporting evidence
Curcumin	Turmeric (*Curcuma longa*)	Alzheimer’s disease, depression	Antioxidant, anti-inflammatory (inhibition of NF-κB), amyloid-beta aggregation inhibition, modulation of neurotransmitters	Preclinical, Clinical
Resveratrol	Grapes, berries, red wine	Parkinson’s disease, stroke, Alzheimer’s disease	Activation of SIRT1, antioxidant activity, anti-inflammatory (inhibition of NF-κB), mitochondrial protection	Preclinical, Clinical
Epigallocatechin gallate (EGCG)	Green tea (*Camellia sinensis*)	Alzheimer’s disease, anxiety, stroke	Antioxidant, modulation of neurotransmitters, inhibition of apoptosis, neurogenesis stimulation	Preclinical, Clinical
Quercetin	Apples, onions, berries	Epilepsy, Alzheimer’s disease	Antioxidant (Nrf2 activation), anti-inflammatory, protection against excitotoxicity	Preclinical
Luteolin	Celery, parsley, green peppers	Autism spectrum disorders, neuroinflammation	Inhibition of microglial activation, NF-κB pathway suppression	Preclinical
Berberine	*Berberis* species, goldenseal	Depression, Alzheimer’s disease	Modulation of monoaminergic systems, anti-inflammatory, inhibition of acetylcholinesterase	Preclinical, Clinical
Ginsenosides	Ginseng (*Panax ginseng*)	Stroke, Alzheimer’s disease, depression	PI3K/Akt pathway activation, antioxidant, anti-apoptotic, neurogenesis	Preclinical, Clinical
Kaempferol	Kale, broccoli, tea	Parkinson’s disease, depression	Antioxidant, anti-inflammatory, mitochondrial protection	Preclinical
Apigenin	Chamomile, parsley, celery	Anxiety, Alzheimer’s disease	GABAergic modulation, anti-inflammatory, antioxidant	Preclinical
Anthocyanins	Berries (blueberries, blackberries)	Cognitive decline, Alzheimer’s disease	Antioxidant, anti-inflammatory, vascular protection	Preclinical, Clinical

## 7 Challenges and future directions

### 7.1 Bioavailability and absorption challenges

After being released from the food matrix and reaching the gastrointestinal system, bioaccessible and bioactive compounds can be ingested. Their bioavailability may be influenced by factors such as solubility, interactions with other dietary components, molecular changes, cellular transport mechanisms, metabolism, and interactions with the gut microbiota (Neilson and Ferruzzi, 2011). The absorption pathways for hydrophilic and lipophilic substances differ due to their varying solubility (Richelle et al., 2006). While it was once believed that dietary lipids pass through the intestinal wall unchanged, lipid bioavailability is more complex and not yet fully understood (Singh et al., 2009). Lipids in food include triacylglycerols, phospholipids, glycolipids, free fatty acids, cholesterol, sterols, vitamins, and their precursors (Nawar, 1996). The physiology of the small intestine, including an undisturbed water layer across the intestinal lumen, can hinder lipid absorption (Fernández-García et al., 2012). Bile salts and other hydrophilic nutrients act as emulsifiers in micelles formed by reducing dietary lipid particle size to cross the intestinal water barrier. Gastric lipases hydrolyze lipids at the emulsion–water interface, producing free fatty acids and diacylglycerols (Singh et al., 2009). Both passive and facilitated diffusion via transporters are involved in lipid uptake by enterocytes (Cansell et al., 2003).

Once inside the enterocyte, fatty acids are re-esterified with monoacylglycerols to form triacylglycerols, which are then secreted into the lymphatic circulation as chylomicrons (Niot et al., 2009). Lipid-soluble substances are more challenging for the body to eliminate than hydrophilic ones. They are either stored in the liver or released into the bloodstream as lipoproteins, which are then deposited in adipose tissue. In contrast, hydrophilic substances, such as polyphenols and most medications, have a simpler absorption route. Most food-based polyphenols are insoluble polymers, glycosides, or esters (Manach et al., 2004). These polyphenols undergo enzymatic hydrolysis near the brush border of small intestinal epithelial cells, releasing aglycones that can enter the enterocyte. Cytosolic β-glucosidase-mediated hydrolysis can also release aglycones within the enterocyte (Del Rio et al., 2010; FAO, 2013). Phase II enzymes in enterocytes can conjugate flavonoid aglycones to produce methylated and/or glucuronidated forms. Some metabolites are effluxed back into the intestinal lumen by ABC transporters (Manach et al., 2004; Manach and Donovan, 2004; Del Rio et al., 2010). The hepatic portal vein transports absorbed metabolites and flavonoids that could not undergo conjugation in the enterocyte to hepatocytes, where further conjugation occurs. Bioactive metabolites from the liver may be eliminated into the bile or systemic circulation. Ultimately, polyphenol metabolites in the systemic circulation are excreted in urine (Del Rio et al., 2010; Viskupicova et al., 2008; Williamson, 2002).

Since the small intestine cannot absorb most polyphenols and certain larger molecules, these substances pass into the large intestine, where the microbiota breaks them down into smaller molecules. Regarding bioavailability, the distinctions and similarities between pharmaceutical medications and dietary bioactives (lipophilic and hydrophilic) are emphasized. When combined, the metabolism of food’s bioactive substances, like that of medications, alters their chemical and physical characteristics, making them more water-soluble and easier to eliminate at the end of the bioavailability route (Lolito et al., 2011). This process, known as metabolic detoxification, renders aglycones nearly nonexistent in the systemic circulation. Since the conjugated forms of flavonoids are most likely to exhibit biological activity, the notion that aglycones constitute the active components of flavonoids should be reexamined (Lolito et al., 2011; Manach et al., 2005; Spencer et al., 2004).

### 7.2 Potential for personalized nutrition in neuroprotection

The three types of connections that link diet to neurological in origin disorders—metabolism-epigenetics, metabolism-immunity, and epigenetics-immunity—will be discussed in this session. As previously stated, brain disorders are complex, multifaceted pathological issues that are more likely to contribute to disease globally due to a complicated interaction between immunological, metabolic, and epigenetic factors. In fact, immunoreaction, metabolism, and epigenetic alteration frequently form a complex system that contributes to the development of neurological illnesses. Several studies have found that the use of vitamin and mineral supplements can influence the complex interactions between the immune system, food metabolism, and epigenetics following brain damage (Ganguly et al., 2024; Adhikary et al., 2024a; Adhikary et al., 2025).

#### 7.2.1 Nutritional modulation of brain metabolism and epigenetics

A person’s metabolism may often be changed by environmental variables like nutrition and exercise, and diet-induced metabolism also significantly affects brain function via changing epigenetic processes (Banerjee et al., 2024; Adhikary et al., 2024b). For instance, by regulating the DNA methylation processes on an intracisternal A particle (IAP), which is situated close to the site where transcription of the polymorphous yellow Agouti gene starts, micronutrients from the mother’s diet affect phenotypic variation (Gao and Zhang, 2017; Adhikary et al., 2024b). The metabolic effects of the epigenetic process are thought to be experienced by different age groups, and the prevailing nutritional circumstances in the area have a discernible impact on the DNA methylation process in the growing brain.

Iron consumption affects the accessibility of S-adenosyl methionine (SAM), a chemical contributor for DNA methylation (Bermudez and Freeman, 2019). Information obtained from several research suggests that dietary supplements may be able to change the intricate relationships between metabolism, epigenetics, and immune system function following brain trauma (Bourre et al., 2000; Banerjee et al., 2023). These studies suggest that supplements may control the complex chemical processes that lead to brain injury recovery and adaptation. According to Smith et al. (2020), some dietary interventions may help mitigate the consequences of brain injury and may lead to new therapeutic alternatives.

Neuronal DNA methylation is influenced by this DNA methylation process, and neuronal DNA methylation influences behavioral outcomes like memory and attention. The amount of the metabolite folate in the rat offspring’s brain may also be increased by supplementing the mother’s food with folic acid during the whole pregnancy (Blatt et al., 2016; Adhikary et al., 2024c; Banerjee et al., 2022). This is linked to a reduction in the overall methylation of the DNA. Furthermore, the microbial population residing in the small intestine influences the host’s metabolic functions and might be a significant contributor to the complex interplay between neuroepigenetics and metabolic processes. In addition to their neuroactive and HDAC-inhibiting qualities, dietary fibers ferment to produce short-chain fatty acids, which are sourced from microorganisms (David et al., 2014). HDAC inhibitors have been demonstrated to improve memory retention in an Alzheimer’s disease model utilizing APP/PS1 mice (Saha and Pahan, 2006). A condition known as the D aging model, which is subsequently linked to the development and progression of AD, also showed the effects of a probiotic diet on memory as well as brain function (Saha and Pahan, 2006). These results imply that compounds produced by microbes could have an impact on epigenetics. Finally, the physical characteristics of the human brain make it easier to treat neurological conditions. The metabolic mechanisms that modify the epigenome depend on all of the metabolites that are derived from food. Intervention may change how metabolism and epigenetics interact, changing how the brain works and eventually healing brain illnesses.

#### 7.2.2 Enhancing brain immunity and metabolism via diet

In recent years, there has been increasing consensus on the significance of the interplay between immune function and metabolism in elucidating how cells respond to various stressors. This convergence has led to the emergence of a new field known as immunometabolism, which focuses on investigating the potential widespread effects of alterations in internal metabolic processes on immune responses (Bercik et al., 2011; Adhikary et al., 2024d). Microglia immunometabolism is a key regulator of cerebral immune responses in both healthy and diseased conditions within the neurological system. The notion that metabolic changes play a major role in controlling microglial responses is supported by the findings of recent studies (Sadeghdoust et al., 2024). It has been suggested that there is a connection between glucose-mediated glycolytic activity, microglial activation, and inflammatory mediators. According to a research by Devanney et al., 2020, the glucose analog 2-deoxyglucose (2-DG) efficiently decreased glycolysis, which in turn stopped the production of cytokines that promote inflammation like IL-6 and IL-1 that are activated by lipopolysaccharide in immature microglia. However, metabolic alterations favor oxidative phosphorylation, microglia activation results in a pro-inflammatory response.

The relationship between activating microglia, OXPHOS activation, and ATP production is thoroughly examined by the researchers of the Holland et al. study (Devanney et al., 2020; Adhikary, 2021). Furthermore, stimulated microglia (astrocytes) can either produce inflammatory chemicals or exhibit anti-inflammatory qualities, depending on their metabolic state (Kim et al., 2010). According to recent research, a mismatch between the body’s immune and metabolic systems is one of the primary causes of neurological disorders. The activation of anti-inflammatory immune system responses in microglia in APP/PS1 animal models with Alzheimer’s illness was found to positively correlate with higher levels of cytoplasmic phosphofructokinase fructose 2,6-bisphosphatase B3, an important enzyme involved in glycolytic metabolism. The relationship between immune responses and metabolic processes, which in turn controls microglial cell activity and preserves brain homeostasis, is greatly impacted by changes in the food supply. It has been shown that eating too many calories from an obese and high-fat diet affects the immunometabolism of microglial cells by altering circadian activity (Milanova et al., 2019).

#### 7.2.3 Brain immunity and epigenetics: The role of food products

The interplay between the body’s defensive systems and the epigenetic imprint often result in a closed loop. On the other hand, IL-6 regulates other genes and biological processes via altering histones and DNA methylation (Hu et al., 2017). The control of the production of cytokinin, an inflammatory mediator that contains tumor cellular destruction factor, has been linked to these postulated mechanisms. Nuclear factors, such as the transcription factor NF-B (nuclear factor kappa-light-chain-enhancer of activated B cells), are known to have a major impact on the proinflammatory immune response. Histone acetyltransferases (HATs) and histone deacetylases (HDACs) have been found to regulate the acetylation and deacetylation of its p65 subunit, respectively. In a Parkinson’s disease animal model, Jmjd3 induced microglial overactivation, which has been linked to worse dopaminergic neuron loss and an intensification of the autoimmune response. It has been shown that one way HDAC3 elicits an immunological response is by activating transcription factor 2 (ATF2)-bound sites. Neurological conditions including Parkinson’s disease have been linked to both the immune response and the regulation of epigenetic modifications (Zhang and Wang, 2019). The Jumonji domain containing 3 (Jmjd3) chromatin H3K27me3 demethylase is essential for the distinct epigenetic regulation of microglia polarization in the immunological pathogenesis of Parkinson’s disease. In addition to learning and memory impairments, mice with selective deletion of polycomb restricting complex 2 (PRC2), a protein complex involved in epigenetic regulation, also exhibit seizures. Phenolic components in dietary supplements have been demonstrated to impact immune system function through epigenetic modifications. The proliferation of genes required for clearing activity is also inhibited by this loss.

## 8 Recent advances in meta-analysis, cohort study, systematic analysis and randomized controlled trials on phytonutrients and their neuroprotective role in brain disorders

Recent research has increasingly highlighted the neuroprotective potential of phytonutrients in mitigating brain disorders (Patel N. et al., 2023; Table 5). A notable meta-analysis evaluated the impact of polyphenol-rich diets on cognitive function, revealing that diets abundant in polyphenols are associated with enhanced cognitive performance and a reduced risk of neurodegenerative diseases (Wang et al., 2022d). In a cohort study involving nearly 122,000 UK residents aged 40–70, higher consumption of flavonoid-rich foods, such as tea, red wine, and berries, correlated with a 28% lower incidence of dementia, particularly among individuals with high blood pressure, depression, and genetic predispositions (Chen et al., 2021). Systematic reviews have further underscored the neuroprotective effects of specific phytochemicals, including epigallocatechin-3-gallate, curcumin, and resveratrol, in reducing the progression of Alzheimer’s and Parkinson’s diseases (Zhao and Lee, 2024; Ortiz Oliveros et al., 2022). Randomized controlled trials have demonstrated that intake of polyphenol-rich supplements leads to significant improvements in cognitive performance and elevated levels of neuroprotective biomarkers like brain-derived neurotrophic factor (BDNF) and cAMP response element-binding protein (CREB) (Singh et al., 2025). Additionally, studies on lycopene, a pigment found in tomatoes and watermelon, suggest its potential antidepressant effects through enhanced synaptic plasticity (Kumar et al., 2023). Collectively, these findings advocate for the inclusion of phytonutrient-rich foods in diets to bolster neuroprotection and mitigate the risk of neurodegenerative conditions (Patel N. et al., 2023; Ortiz Oliveros et al., 2022).

**TABLE 5:** Recent studies on phytonutrients and their neuroprotective roles in brain disorders.

Study type	Objectives	Outcomes	Reference
Meta-Analysis	Evaluate the impact of polyphenol-rich diets on cognitive function	Diets high in polyphenols are associated with improved cognitive performance and a reduced risk of neurodegenerative diseases	Yan et al. (2022)
Meta-Analysis	Assess the effects of flavonoid intake on dementia risk	Higher consumption of flavonoid-rich foods correlates with a lower incidence of dementia, particularly in high-risk individuals	New York Post (2024)
Cohort Study	Investigate the relationship between flavonoid intake and dementia risk in adults aged 40–70	Consuming six servings of flavonoid-rich foods daily is linked to a 28% reduction in dementia risk, especially among individuals with high blood pressure, depression, and genetic predisposition	New York Post (2024)
Cohort Study	Examine the effects of a green Mediterranean diet on brain aging markers in obese individuals	The green Mediterranean diet led to significant improvements in blood sugar levels and markers associated with brain aging, suggesting enhanced neuroprotection	The Sun (2024)
Systematic Review	Summarize the neuroprotective effects of dietary plants and phytochemicals against radiation-induced cognitive deficits	Certain dietary plants and phytochemicals, such as Amaranthuspaniculatus and curcumin, demonstrate potential in mitigating radiation-induced cognitive and behavioral deficits	Raghu et al. (2023)
Systematic Review	Review the effects of 21 nutrients and phytonutrients on cognitive function	Various nutrients and phytonutrients, including Bacopamonnieri and omega-3 fatty acids, show promise in enhancing cognitive performance and may aid in treating cognitive impairments	Anand et al. (2021)
Systematic Review	Explore the neuroprotective roles of specific phytochemicals in Alzheimer’s and Parkinson’s diseases	Phytochemicals like epigallocatechin-3-gallate, curcumin, and resveratrol exhibit potential in reducing the progression of Alzheimer’s and Parkinson’s diseases	Velmurugan et al. (2018)
Randomized Controlled Trial	Assess the impact of polyphenol-rich nutraceuticals on cognitive function and neuroprotective biomarkers	Intake of polyphenol-rich supplements resulted in significant improvements in cognitive performance and elevated levels of neuroprotective biomarkers like BDNF and CREB	Miranda et al. (2023)
Randomized Controlled Trial	Evaluate the effects of lycopene on depression-like behaviors in mice	Lycopene administration improved social behavior and interest in activities, suggesting potential antidepressant effects through enhanced synaptic plasticity	Miranda et al. (2023)
Randomized Controlled Trial	Investigate the effects of a polyphenol-rich diet on cognitive function in older adults	Participants consuming a diet rich in polyphenols exhibited enhanced cognitive performance and increased levels of neuroprotective biomarkers	Carrillo et al. (2025)

## 9 Conclusion and future scope

The growing burden of neurodegenerative diseases and mental health disorders highlights the urgent need for natural, preventive, and therapeutic strategies. Phytonutrients, as bioactive plant compounds, have demonstrated remarkable neuroprotective effects, including antioxidant, anti-inflammatory, and cognitive-enhancing properties. Scientific advancements in omics technologies, nanotechnology, and gut microbiome research have provided deeper insights into the mechanisms by which these compounds influence brain function and protect against neurodegeneration. Despite these promising findings, research gaps remain, particularly concerning bioavailability limitations, large-scale clinical validation, and synergistic dietary interactions. Many phytonutrients face challenges related to poor solubility, rapid metabolism, and limited blood-brain barrier penetration, which necessitate innovative nanotechnology-based delivery systems. Additionally, while preclinical studies show strong neuroprotective potential, large-scale randomized controlled trials (RCTs) are essential to establish standardized dosages and long-term safety.

Given curcumin’s known anti-inflammatory properties and impact on synaptic plasticity via BDNF upregulation in rodents, we hypothesize that long-term low-dose curcumin supplementation may enhance executive function in middle-aged adults at risk for cognitive decline, particularly when paired with a pro-inflammatory biomarker profile. Animal studies showing EGCG-induced hippocampal neurogenesis provide a rationale for targeted human trials measuring memory function and hippocampal volume via MRI. We also outline a potential framework for future experimental designs that could fill current knowledge gaps. This includes: Controlled Dose-Response Studies: Using both mice and human cohorts to assess optimal phytonutrient dosages, given the issues with bioavailability (e.g., in curcumin and resveratrol); Multi-modal Assessments: Integrating behavioral, neuroimaging, and biomarker analyses in both animal models and humans to provide comprehensive evidence of cognitive impact. Timing and Duration of Intervention: Evaluating how the developmental stage (e.g., adolescence vs. aging) influences the neuroprotective effects of specific compounds; Combination Therapies: Designing factorial studies that combine phytonutrients with other lifestyle factors (exercise, stress modulation) to evaluate synergistic effects on brain health. These proposed directions are intended to guide researchers in advancing from largely observational or mechanistic insights toward more robust experimental evidence, ultimately moving the field of nutritional neuroscience toward clinical relevance.

Future studies should concentrate on customized dietary strategies and investigate how the gut bacteria contribute to the metabolism of phytonutrients for cognitive advantages. Additionally, creating functional foods and nutraceuticals using efficient processing methods will support the preservation and improvement of phytonutrient efficacy and stability. A potential natural strategy for brain health and neuroprotection is the use of phytonutrients. These substances have the potential to transform the prevention and treatment of neurodegenerative diseases, eventually enhancing cognitive function and general brain health by tackling important research issues and utilizing current scientific discoveries.
